# Constrictive Pericarditis: A Diagnostic Conundrum

**DOI:** 10.7759/cureus.39485

**Published:** 2023-05-25

**Authors:** Aryan Mehta, Mridul Bansal, Yashashwi Pokharel, Saraschandra Vallabhajosyula

**Affiliations:** 1 Section of Cardiovascular Medicine, Department of Medicine, Wake Forest School of Medicine, Winston-Salem, USA

**Keywords:** cardiac imaging, constrictive pericarditis, interventional cardiology, heart failure, pericardial disease

## Abstract

A 65-year-old male presented with chest pain, tachycardia, tachypnea, and diminished breath sounds. His lab investigations revealed an elevated leukocyte count, erythrocyte sedimentation rate, and B-type natriuretic peptide. Transthoracic echocardiography and chest imaging revealed the presence of pericardial effusion without tamponade and he was treated for presumed acute idiopathic pericarditis. He was started on indomethacin and colchicine but he stopped them prematurely due to side effects. Subsequently, he developed pleural effusions and ascites requiring multiple thoracenteses and paracenteses. Due to equivocal echocardiographic findings, he underwent invasive hemodynamic measurements which demonstrated equalization of filling pressures and ventricular interdependence, confirming constrictive pericarditis. Due to ongoing pericardial inflammation on cardiovascular magnetic resonance imaging, he was treated with a prednisone taper. Due to persistent symptoms and fibrosis of the pericardium on cross-section imaging, he underwent pericardiectomy. He did well with the procedure and has had an uneventful clinical follow-up.

## Introduction

Constrictive pericarditis (CP) is uncommonly seen in the United States and is often due to undiagnosed or under-treated pericarditis [1]. Idiopathic or post-viral pericarditis is the most common cause of CP [2]. Due to the non-specific symptoms, insidious onset, and no clear temporal evolution, this presents as a diagnostic challenge. Echocardiography is the first-line imaging modality for the diagnosis and invasive hemodynamic measurements are reserved for ambiguous cases [3]. Pericardiectomy remains the definitive treatment for CP, however, a trial of anti-inflammatory medications is preferred initially [4]. We present a case of inadequately treated acute pericarditis resulting in constriction necessitating pericardiectomy.

## Case presentation

**Figure 1:**
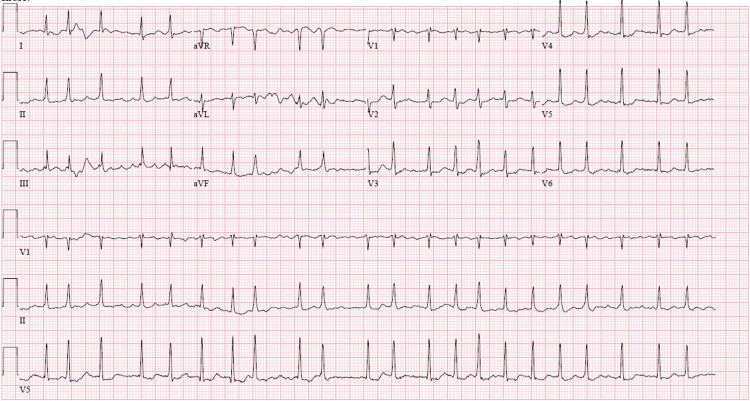
Electrocardiogram at presentation

A 65-year-old male with known essential hypertension, diabetes mellitus type 2, hyperlipidemia, and tobacco abuse presented for the evaluation of right-sided chest pain which was progressive over 4 days. The pain was described as dull aching pain in the chest, radiating to the neck and right shoulder with an associated acute onset of progressive fatigue and dyspnea on moderate to severe exertion. On examination, he had a heart rate of 121 beats/minute, respiratory rate of 24/minute, and oxygen saturation of 97% on room air. On auscultation, he had diminished breath sounds on the right lower lung zones with normal cardiac auscultation. At the time of presentation, his laboratory investigations revealed an elevated leukocyte count (12.7 x 10³/սL), erythrocyte sedimentation rate (ESR) (68 mm/hour), C-reactive protein (CRP) (111 mg/L) and B-type natriuretic peptide (BNP) (326 pg/ml). Tests for novel coronavirus 2019, influenza A/B, and rheumatic disease (rheumatoid factor and antinuclear antibody) were all negative. Troponins at this time were not elevated. His electrocardiogram (ECG) demonstrated sinus tachycardia with no PR segment abnormalities. However prolonged QTc, non-specific ST-segment, and T-wave abnormalities were noted (Figure 1).

Chest imaging revealed a small right pleural effusion with overlying atelectasis, a small to moderate-sized pericardial effusion, and no evidence of pulmonary embolus. Transthoracic echocardiography (TTE) demonstrated a left ventricular ejection fraction (LVEF) of 65-70% with a moderate-sized pericardial effusion without evidence of cardiac tamponade. Other findings included trace tricuspid regurgitation and mildly dilated inferior vena cava (IVC) with no inspiratory collapse. No pleural effusion was seen. During the hospitalization, his leucocytosis resolved and the patient developed paroxysmal atrial fibrillation which auto-converted to normal sinus rhythm. After ruling out other etiologies, the symptoms were considered secondary to suspected viral/inflammatory pericarditis. The patient was discharged on indomethacin (50 mg thrice daily) and colchicine (0.6 mg twice daily). The patient was discharged on indomethacin and colchicine. Two weeks after dismissal, due to concerns for diarrhea, the patient stopped his colchicine medication. His indomethacin was discontinued one month later due to his anemia and abnormal kidney function.

**Figure 2:**
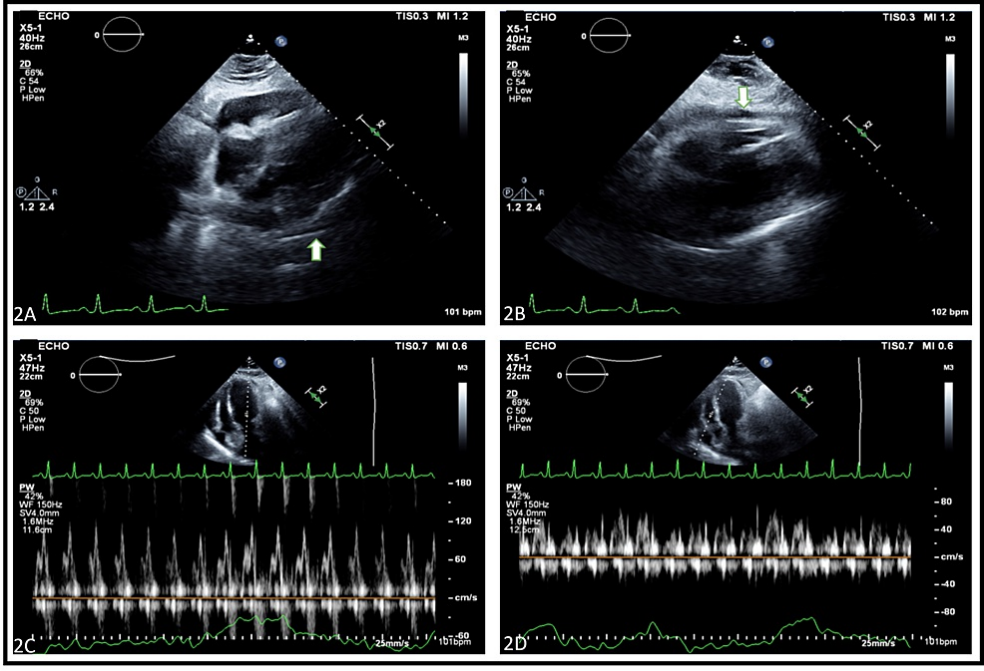
Transthoracic echocardiogram at presentation demonstrating moderate anteriorly location pericardial effusion (2A and 2B) without evidence of significant respiratory variation of inflow velocities across the mitral (2C) and tricuspid (2D) valves

The patient presented four months later with complaints of shortness of breath, abdominal distension, and edema. His laboratory investigations at this time revealed a BNP of 225 pg/ml, CRP of 60.7 mg/L, and ESR of 26 mm/hr. A repeat computerized tomography (CT) scan done during this time revealed increased pericardial thickness without calcifications, mild smooth right pleural thickening, moderate left and small dependent right pleural effusions, and small volume ascites. TTE at this point noted an LVEF of 55-60% with a moderate pericardial effusion. It also showed some respiratory variation of the Doppler flow across the cardiac valves and dilation of IVC, both of which were concerning for pericardial constriction, but not diagnostic (Figure 2A-2D).

**Figure 3:**
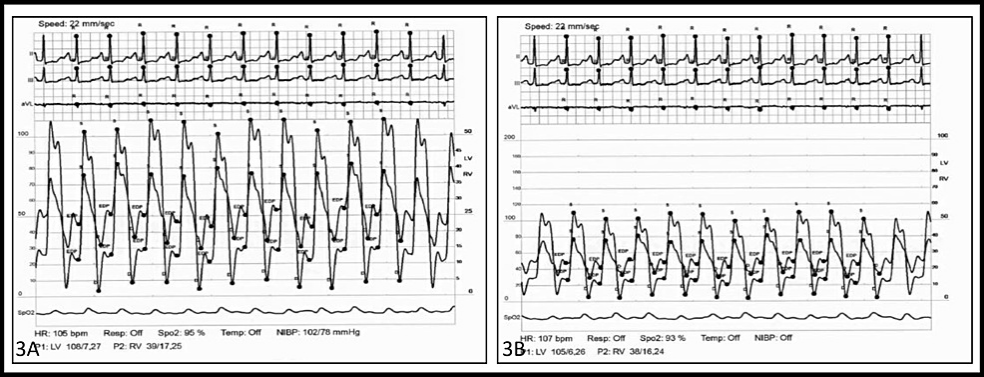
Simultaneous left and right heart catheterization demonstrating elevated end-diastolic pressures, equalization of biventricular pressures, and interventricular dependence with intrathoracic disassociation pathognomic for constrictive pericarditis

Given the inconclusive and limited echocardiographic findings, he underwent invasive hemodynamic measurements. Simultaneous right and left catheterization demonstrated the following pressures: right atrium 25 mmHg, right ventricle 39/14 (mean 24) mmHg, pulmonary artery 42/28 (33) mmHg, pulmonary capillary wedge pressure 25 mmHg, and left ventricular pressure 107/5 (23) mmHg. His cardiac output was 4.2 L/min and his cardiac index was 1.8 L/min/m2. Due to elevated right and left atrial filling pressures, equalization of filling pressure in all the chambers, ventricular interdependence, and decreased cardiac output, a confirmatory diagnosis of constrictive pericarditis was made (Figure 3A-3B).

**Figure 4:**
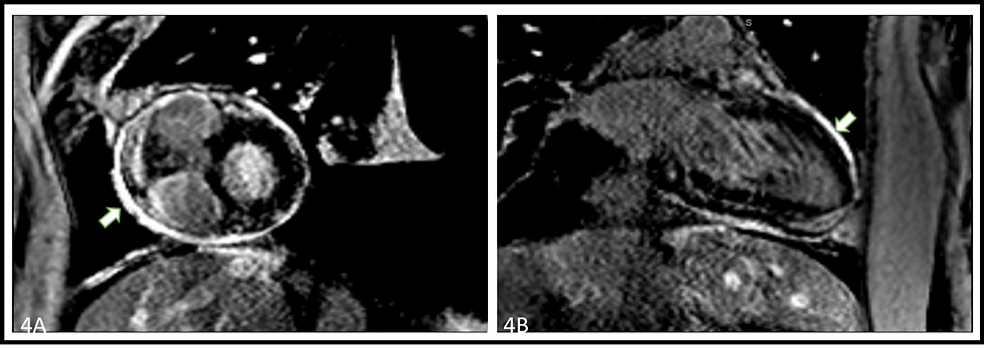
Cardiovascular magnetic resonance imaging mono-images demonstrating late gadolinium pericardial enhancement suggestive of active pericardial inflammation

However, he had multiple hospitalizations over the course of four months for volume overload and received extensive diuresis, and multiple therapeutic thoracenteses and paracenteses. The patient was re-challenged with another trial of medical therapy using ibuprofen and colchicine. The patient continued to have diarrhea from colchicine therapy and could not continue ibuprofen due to acute kidney injury. A cardiovascular magnetic resonance (CMR) imaging done 4 weeks after restarting medical therapy depicted small pericardial effusion with circumferential pericardial enhancement and ventricular interdependence with respiration suggestive of constrictive pericarditis with ongoing pericardial inflammation (Figure 4A-4B). An enlarged left atrium and a large left-sided pleural effusion were also seen. Due to the presence of continued inflammation of the pericardium and failure to tolerate the second round of medical therapy, he was transitioned to a prolonged prednisone taper.

**Figure 5:**
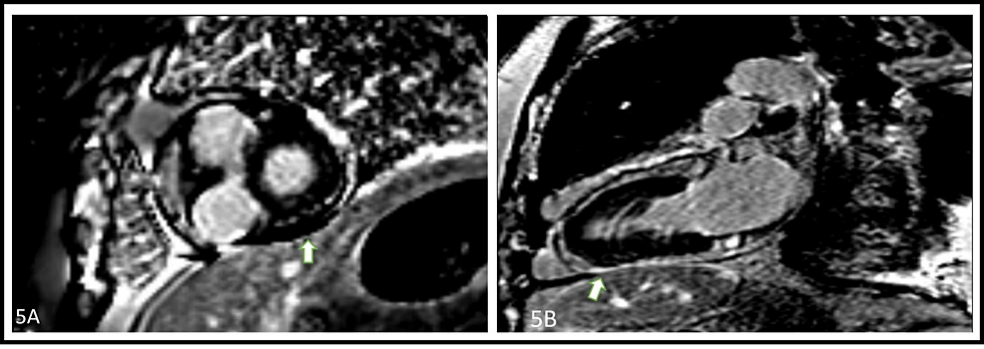
Repeat cardiovascular magnetic resonance imaging mono-images demonstrating limited to absent late gadolinium pericardial enhancement suggestive of limited pericardial inflammation

Three months after this presentation, he developed intermittent shortness of breath. EKG demonstrated new-onset atrial flutter with a rapid ventricular response and he was admitted to the hospital for further management. He received cautious diuresis, amiodarone therapy, and anticoagulation. He auto-converted to normal sinus rhythm and restarted on a higher dose of prednisone with the intent to gradually taper the medication. Repeat CMR demonstrated pericardial thickening with no pericardial enhancement. The LVEF was 73% and there was no evidence of ventricular interdependence (Figure 5A-5B). As the CMR did not show any further evidence of pericardial inflammation, he was planned for a pericardiectomy. 

Investigations

Intra-operative pre-intervention trans-esophageal echocardiography (TEE) revealed normal right ventricular function, LVEF of >55%, mild to moderate mitral regurgitation, and trace pulmonary and tricuspid regurgitation. No aortic regurgitation was seen. It also showed a large left pleural effusion. Intra-operative post-intervention TEE revealed an LVEF of 65-70% with trace mitral and tricuspid regurgitation. No aortic or pulmonary regurgitation was seen. 

Treatment

The patient underwent a pericardiectomy via median sternotomy. The pleura was mobilized bilaterally off the pericardium after which a left-sided moderate to large pleural effusion was evacuated. The pericardium was opened in the midline with a T-incision along the diaphragm. The pericardium was then mobilized off the epicardium and after exposing various structures, the left portion of the pericardium was removed down to the level just above the phrenic nerve, followed similarly by the right. Two drains were placed in the mediastinum and one in the left pleural space which were removed later. On histopathological examination, pericardial tissue showed fibro-adipose tissue with scattered lymphoplasmacytic inflammation. No active inflammation or malignancy was identified.

Outcome and follow-up

The patient was extubated on the day of surgery. His vitals were stable and clear bilateral breath sounds were heard on auscultation. He developed post-operative atelectasis for which the use of incentive spirometry was encouraged. The remainder of the postoperative hospitalization course was uneventful. TTE done just before discharge noted normal left ventricular size, hyperdynamic left ventricle, LVEF of 65-70%, and no significant valvular stenosis or regurgitation. He was discharged on postoperative day six on home physical therapy. The steroid taper was then started again, which was completed two weeks later. Additionally, he was continued on diuretics, amiodarone, and anticoagulation.

## Discussion

The true prevalence of CP is not very well known [5]. In the developed world, idiopathic pericarditis is the most common cause of CP while infectious etiology is the most common cause in developing nations [1]. Other important causes are post-treatment pericarditis, viral infection, and connective tissue disorders among many others. In a prospective study, 500 patients who had their first episode of pericarditis were studied to evaluate the evolution toward CP. Of the 500 patients, 416 had pericarditis due to viral/idiopathic etiology and 84 due to nonviral/non-idiopathic etiology. Two of the 416 (0.48%) patients with viral/idiopathic etiology versus seven of the 84 (14.5%) patients with nonviral and non-idiopathic etiology developed CP [6].

CP should be distinguished from other causes of right-sided heart failure such as pulmonary embolism, pulmonary hypertension, right ventricular infarction, and mitral stenosis [7]. Echocardiography is the first-line imaging modality for the evaluation of pericardial diseases [8]. Increased pericardial thickness noted on CT and CMR can be used to distinguish between CP and restrictive cardiomyopathy [8]. Even with high-quality cardiac imaging, cardiac catheterization and hemodynamic testing remain the gold standard for its diagnosis, especially in equivocal cases. CP and restricted cardiomyopathy share the same pathophysiologic elements and can be diagnosed and differentiated using hemodynamic catheterization. For their diagnosis, elevation of diastolic pressures, low cardiac output or both are needed. Useful diagnostic parameters for CP include respirophasic variation in pressures and stroke volume, enhanced ventricular interdependence, equalization of intracardiac diastolic pressures, and dissociation of intrathoracic and intracardiac pressures [3].

The medical management for idiopathic/viral pericarditis includes treatment with non-steroidal anti-inflammatory drugs (NSAIDs), colchicine, and corticosteroids. The most commonly used NSAIDs are aspirin, ibuprofen, indomethacin, and ketorolac [9]. In addition to NSAIDs, the COPE trial showed the effectiveness of adding colchicine in managing such patients effectively [10]. For those who have contraindications or have failed initial medical therapy with NSAIDs and colchicine, corticosteroids can be administered [9, 10]. Surgical treatment with pericardiectomy may be required in patients who fail medical management, as noted in our case. Pericardiectomy carries high mortality and morbidity rates and remains the definitive treatment for chronic CP as it provides symptomatic relief and significant improvement in functional status [4]. Busch et al reported reduced LVEF and right ventricular dilatation to be independent predictors for early mortality, and coronary artery disease, chronic obstructive pulmonary disease, and renal insufficiency as risk factors for late mortality [11]. They also described the superiority of partial pericardiectomy over total pericardiectomy, even though it is refuted by various studies.

## Conclusions

This study highlights the predicament in diagnosing and treating constrictive pericarditis. Invasive hemodynamic measurements, which are the gold standard of diagnosis, are useful when echocardiographic findings are equivocal. Constrictive pericarditis closely mimics restrictive cardiomyopathy and needs to be clearly distinguished prior to therapy. Steroids are used as second-line agents for inadequately treated chronic pericarditis. Pericardiectomy carries a high mortality risk and is definitive treatment of constrictive pericarditis. A high index of suspicion, early diagnosis, and immediate therapeutic interventions are required to facilitate recovery and reduce recurrence.
